# Supplementary material for: Resolving inter-regional communication capacity in the human connectome
Source: Netw Neurosci. 2023 Oct 1;7(3):1051–79. doi: 10.1162/netn_a_00318 (PMC10473316; doi:10.1162/netn_a_00318)
Supplement: Supplementary file 1 [file netn-7-3-1051-s001.pdf]

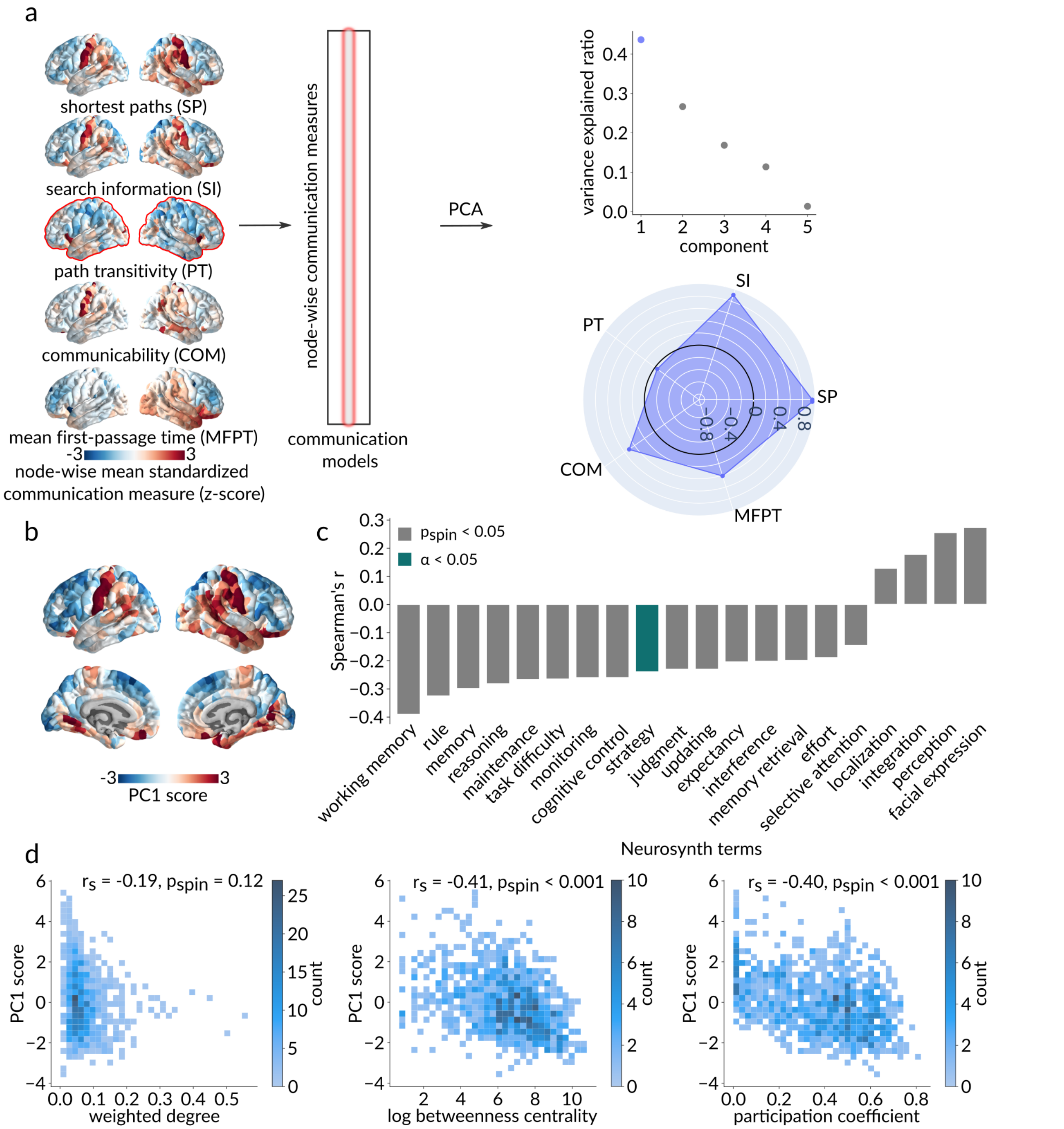

Figure S1. **Extending to multiple communication models - node-level** | (a) Standardized node-wise communication measures across five communication models were assembled into the columns of a nodes $\times$ communication models matrix. Principal component analysis, applied to this matrix, identified a single dominant component that accounts for 43.65% of the node-level variance in communication capacity. The radar chart represents the first component's loadings (i.e., correlations with the five communication models under consideration), with the greatest contribution to the aggregated communication measure coming from search information and path length, with only a minor contribution from path transitivity. (b) Brain map of PC1 scores (c) Significant anticorrelations ($p_{\text{spin}} < .05$ in grey; Bonferroni corrected, $\boldsymbol{\alpha} = \mathbf{.05}$ in green) between node-wise mean standardized path lengths and Neurosynth functional activation maps associated to higher-order cognitive functions were replicated using PC1 scores. (d) Relationships between PC1 scores and topological features of the empirical weighted structural network recapitulate results obtained using node-wise standardized path lengths. No significant relationship was found with weighted degree ($r_s = -.19$, $p_{\text{spin}} = .12$; left), while a significant negative Spearman correlation was found between PC1 score and betweenness ($r_s = -.41$, $p_{\text{spin}} < .001$; middle) and participation ($r_s = -.40$, $p_{\text{spin}} < .001$; right).

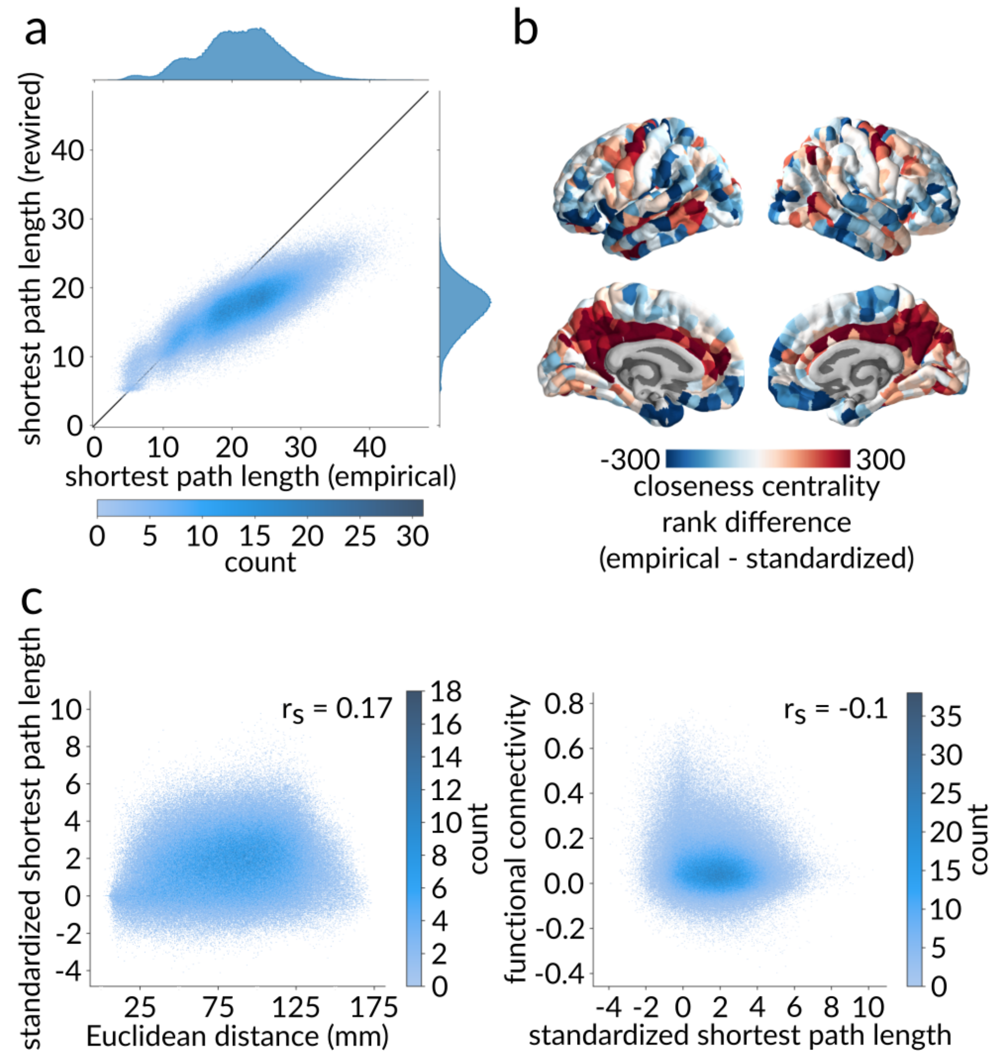

Figure S2. **Disentangling the contributions of topology and geometry |** (a) Scatter plot between empirical (abscissa) and rewired (ordinate) shortest path lengths obtained using a geometry-preserving null model, where each point represents a pair of brain regions. Marginal distribution histograms are shown on the top and right axes. Points that appear below the identity line correspond to paths with a shorter length in the rewired networks than in the empirical network, and vice versa for points above the identity line. (b) Brain map of the region-wise differences between rank-transformed closeness centrality (inverse mean path length to the rest of the network) computed using empirical and standardized shortest path lengths. Red regions are more integrated in the empirical network, and blue regions are more integrated in the standardized network. (c) Relationships between standardized shortest path length and Euclidean distance (left) and functional connectivity (right). As expected due to the edge length-preserving surrogate model, the relationship between standardized shortest path length and Euclidean distance is considerably attenuated compared to the result obtained using strictly topology-preserving nulls. The relationship between standardized shortest path length and functional connectivity is maintained ($r_s = -.1$, $p \approx 0$), but is not exponential anymore.

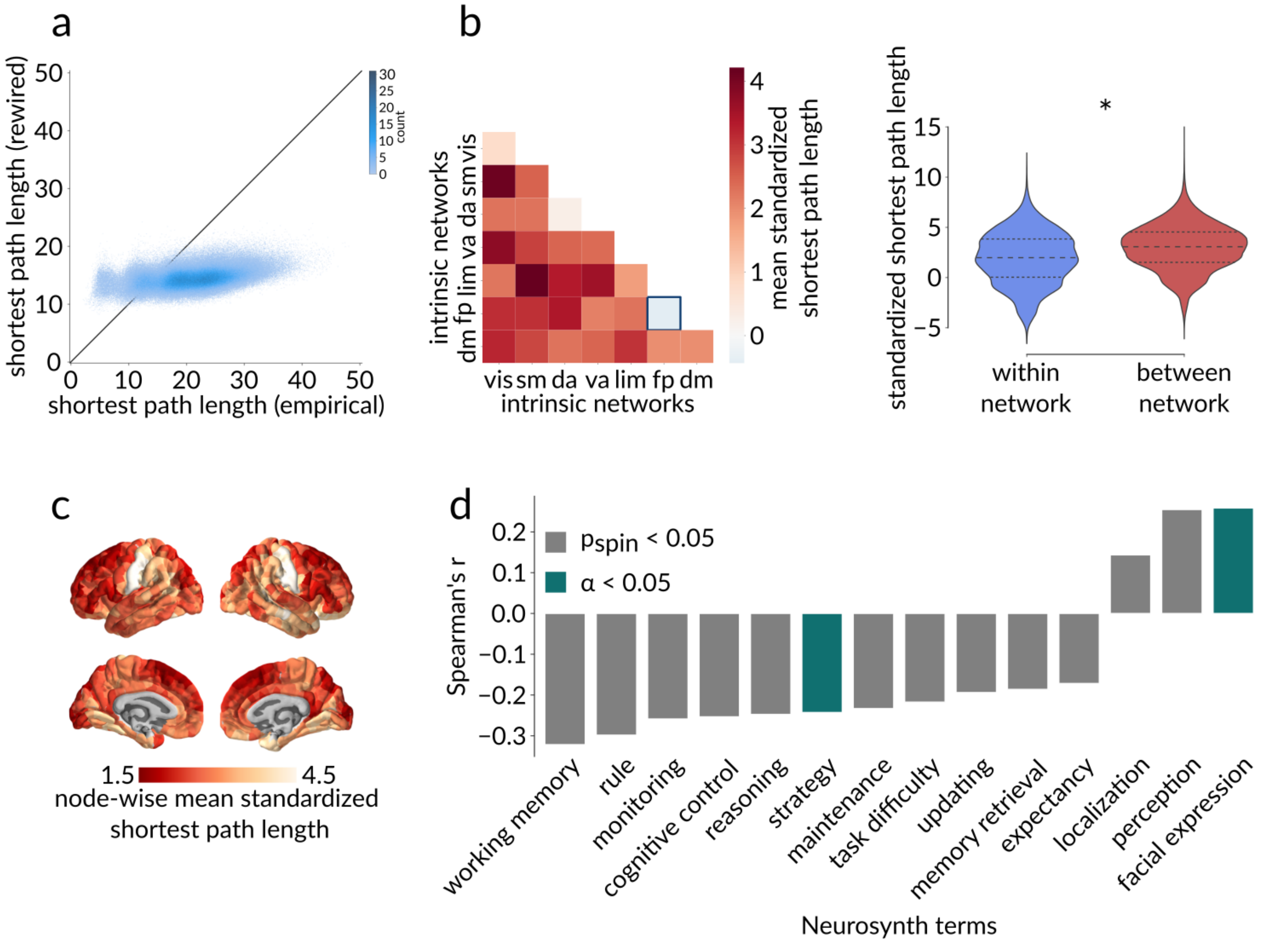

Figure S3. **Sensitivity analysis - *Validation* dataset |** (a) Scatter plot between empirical (abscissa) and rewired (ordinate) shortest path lengths obtained in the *Validation* sample, where each point represents a pair of brain regions. (b) Left: Heatmap of the mean standardized path lengths across node pairs belonging to the same intrinsic network (diagonal) and to different intrinsic networks (off-diagonal). The frontoparietal network's greater-than-expected internal communication capacity is replicated in the *Validation* dataset. Right: The mean within-network standardized path length is also significantly shorter than the mean between-network standardized path length in the *Validation* dataset ($p_{\text{spin}} < .001$). (c) Brain map of mean standardized path length from each node to the rest of the network from the *Validation* set, with red denoting a greater integration of the node within the network and yellow denoting a lower integration. (d) Significant anticorrelations ($p_{\text{spin}} < .05$ in grey; Bonferroni corrected, $\boldsymbol{\alpha = .05}$ in green) between node-wise mean standardized path length and Neurosynth functional activation maps associated to higher-order cognitive functions were replicated in the *Validation* dataset.

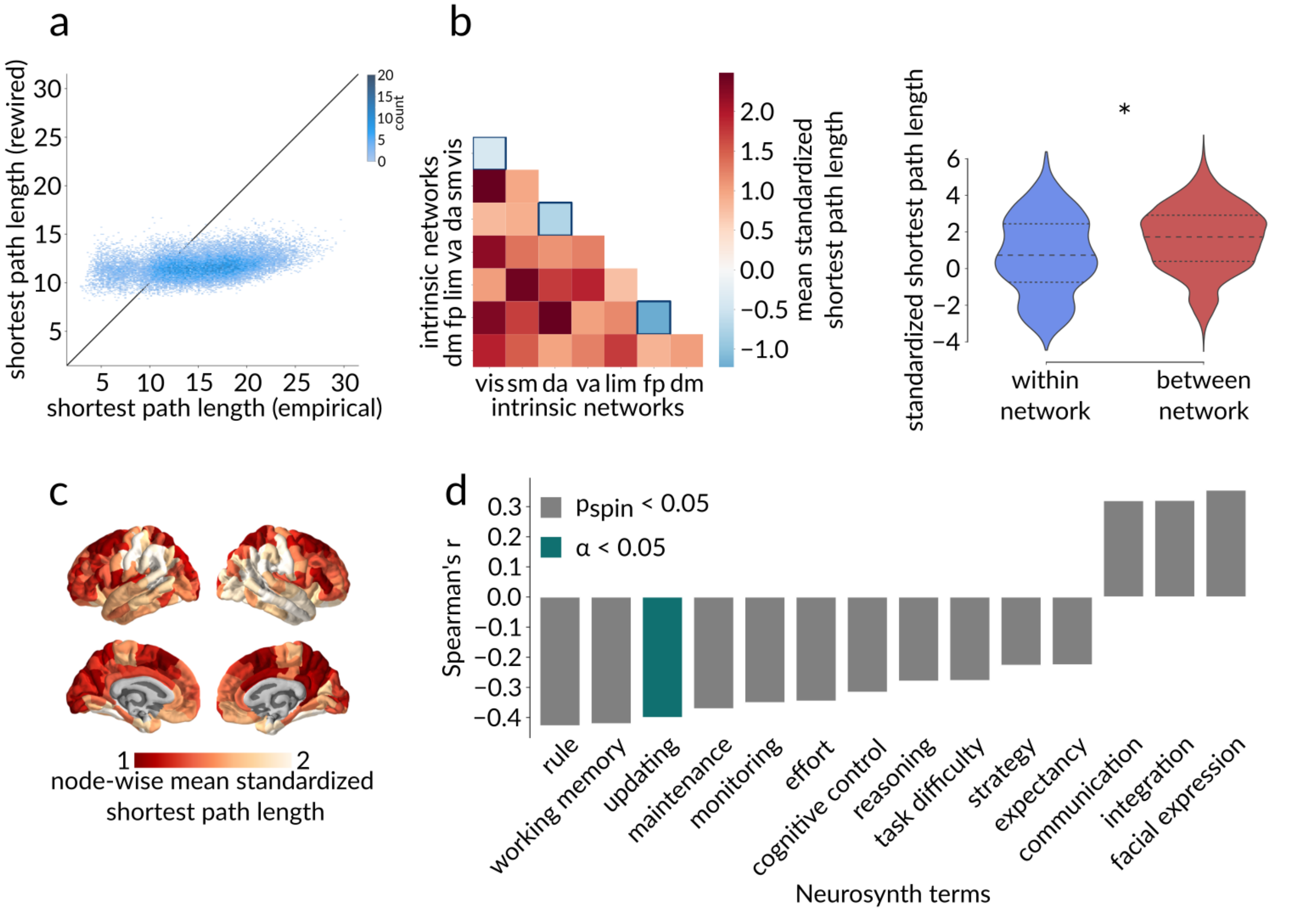

Figure S4. **Sensitivity analysis - 219 nodes resolution |** (a) Scatter plot between empirical (abscissa) and rewired (ordinate) shortest path lengths obtained using the lower resolution Cammoun parcellation, where each point represents a pair of brain regions. (b) Left: Heatmap of the mean standardized path lengths across node pairs belonging to the same intrinsic network (diagonal) and to different intrinsic networks (off-diagonal). In addition to replicating the frontoparietal network's greater-than-expected internal communication capacity, this partition also identifies the communication pathways internal to the visual and the dorsal attention networks as displaying greater-than-expected efficiencies. Right: The mean within-network standardized path length is also significantly shorter than the mean between-network standardized path length when using the 219 nodes resolution of the Cammoun atlas ($p_{\text{spin}} < .001$). (c) Lower resolution brain map of mean standardized path length from each node to the rest of the network, with red denoting a greater integration of the node within the network and yellow denoting a lower integration. (d) Significant anticorrelations ($p_{\text{spin}} < .05$ in grey; Bonferroni corrected, $\boldsymbol{\alpha = .05}$ in green) between node-wise mean standardized path length and Neurosynth functional activation maps associated to higher-order cognitive functions were replicated at a lower resolution.

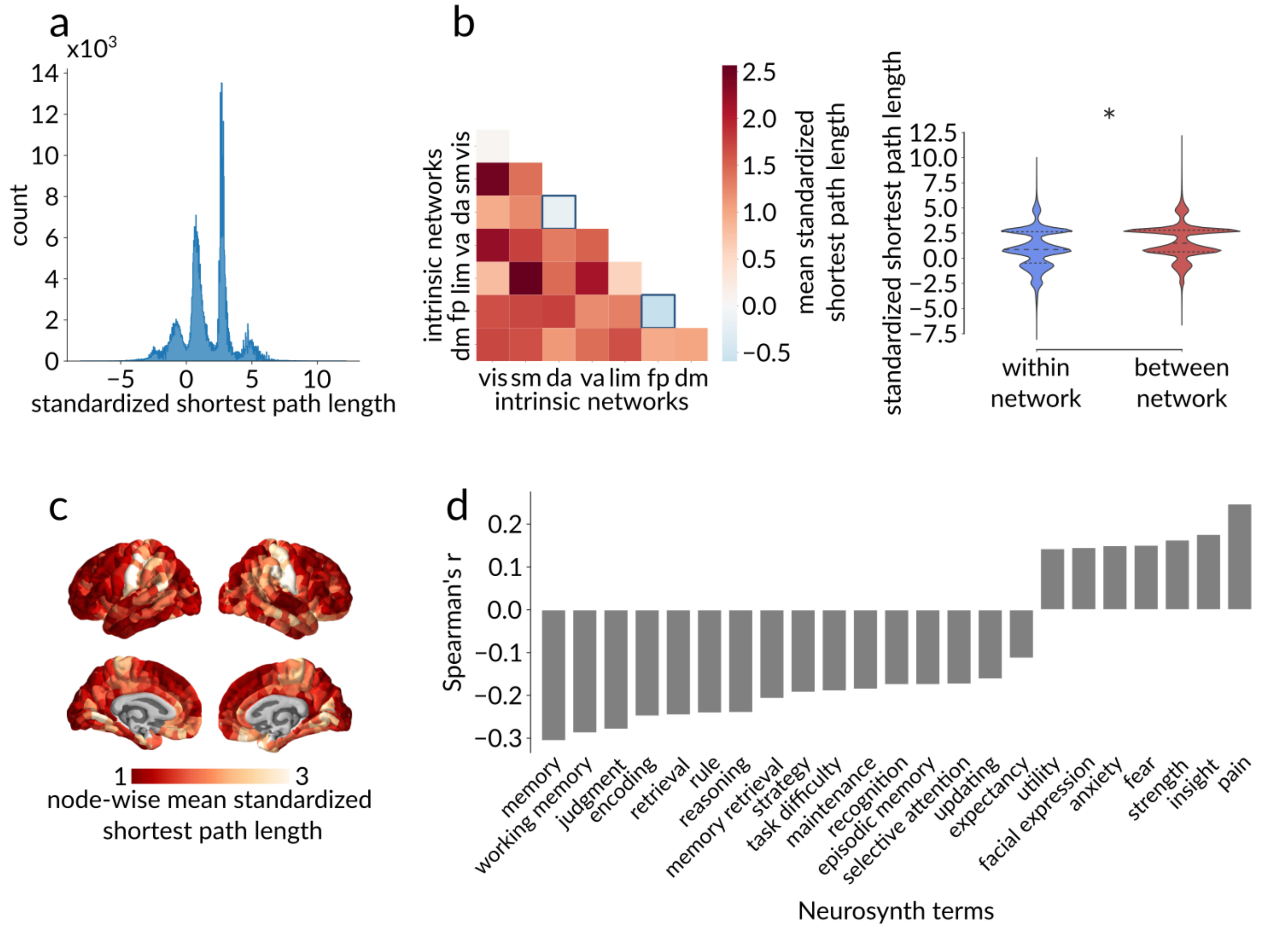

Figure S5. **Sensitivity analysis - binary structural networks |** (a) Distribution of standardized binary shortest path lengths (z-scores) computed from a binary group-consensus structural network for all pairs of brain regions. (b) Left: Heatmap of the mean standardized path lengths across node pairs belonging to the same intrinsic network (diagonal) and to different intrinsic networks (off-diagonal). In addition to replicating the frontoparietal network's greater-than-expected internal communication capacity, binary path lengths also identify greater-than-expected communication capacity in the dorsal attention network. Right: The mean within-network standardized path length is also significantly shorter than the mean between-network standardized path length when using binary structural networks ($p_{\text{spin}} < .001$). (c) Brain map of mean standardized binary path length from each node to the rest of the network, with red denoting a greater integration of the node within the network and yellow denoting a lower integration. (d) Significant anticorrelations ($p_{\text{spin}} < .05$) between node-wise mean standardized path length and Neurosynth functional activation maps associated to higher-order cognitive functions were replicated using binary structural networks.

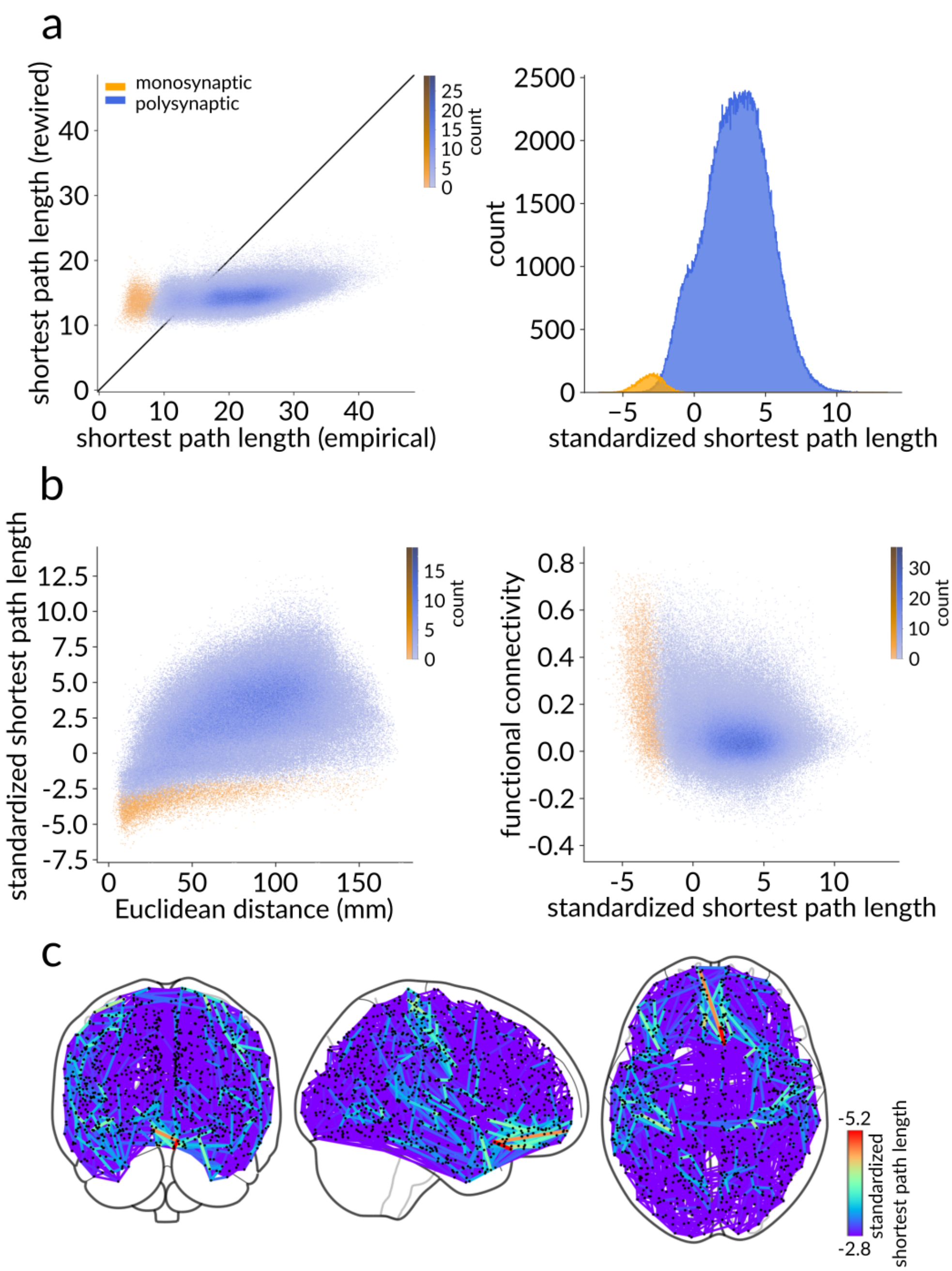

Figure S6. **Sensitivity analysis - Polysynaptic communication pathways |** (a) Left: Scatter plot between empirical (abscissa) and rewired (ordinate) shortest path lengths. Each point represents a pair of brain regions. Right: Distribution of standardized shortest path lengths (z-scores) for all pairs of brain regions. As expected, a large proportion of negative standardized path lengths are attributed to monosynaptic pathways. Monosynaptic communication pathways appear in yellow, whereas polysynaptic shortest paths are coloured in blue. (b) Relationships between standardized shortest path length and Euclidean distance (left) and functional connectivity (right), with monosynaptic connections identified in yellow and polysynaptic paths in blue. (c) Spatial distribution of the top 1% unexpectedly short polysynaptic path lengths

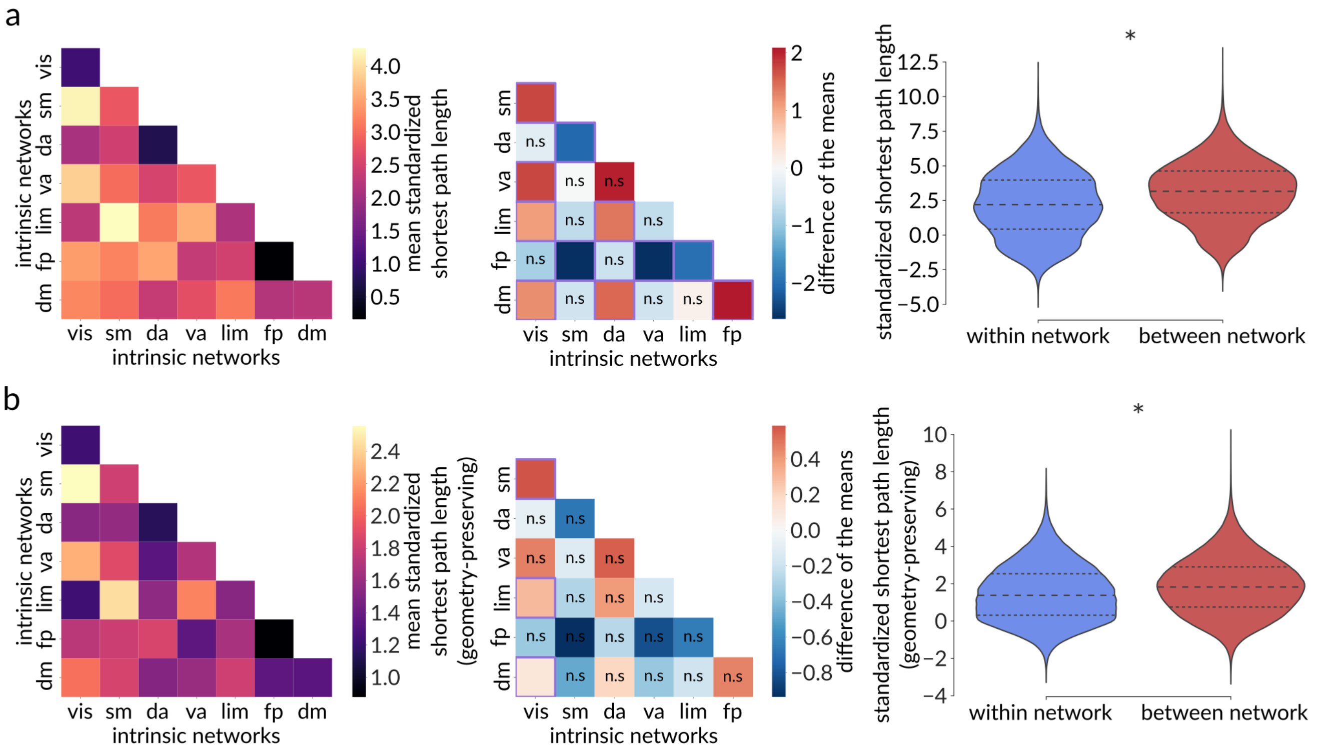

Figure S7. **Sensitivity analysis - Polysynaptic communication pathways delineate functional systems |** (a) Left: Heatmap of the mean standardized path lengths across node pairs belonging to the same intrinsic network (diagonal) and to different intrinsic networks (off-diagonal). In comparison to the previous results considering all communication pathways, including monosynaptic connections, the frontoparietal network's mean standardized path length is no longer negative, suggesting that its greater-than expected internal communication capacity is partly due to highly efficient direct anatomical connections. Middle: Heatmap of the pairwise differences of the means among Yeo intrinsic networks, calculated as the mean value of the network on the y-axis minus the mean value of the network on the x-axis, with the mean value corresponding to the mean standardized path length across node pairs belonging to the same network (diagonal elements of the left heatmap). A purple square indicates significant difference of the means based on network label permutation using spatial autocorrelation-preserving null models (Bonferroni corrected, $\boldsymbol{\alpha = .05}$), whereas "n.s." denotes not significant differences. This plot recapitulates the results obtained when taking all communication pathways into account, with the frontoparietal network displaying the highest internal communication capacity and the somatomotor network exhibiting the lowest internal communication capacity. Right: The mean within-network standardized path length is also significantly shorter than the mean between-network standardized path length when considering only polysynaptic communication pathways ($p_{\text{spin}} < .001$). (b) Same as (a) but for polysynaptic communication pathways standardized using a geometry-preserving null model.

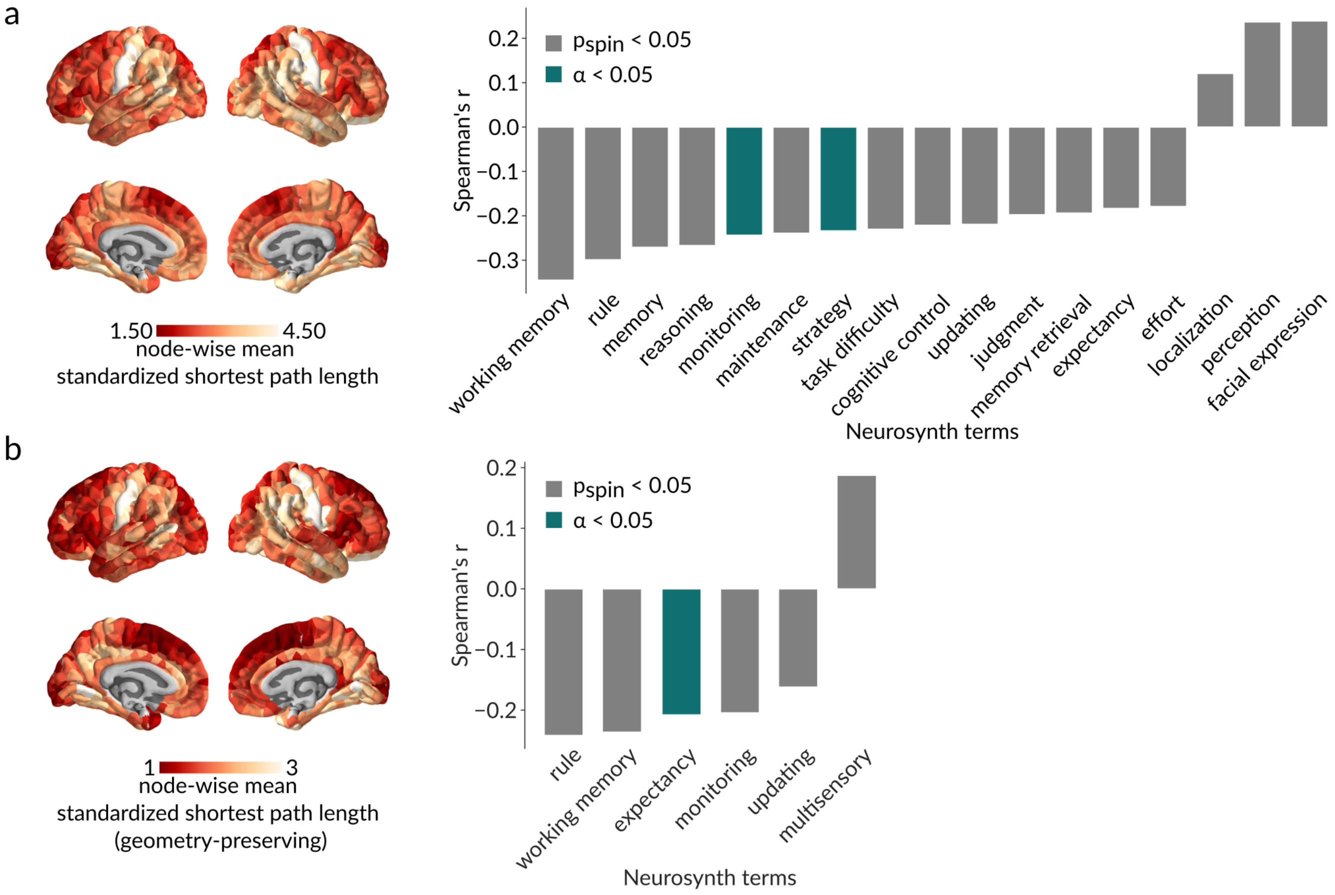

Figure S8. **Sensitivity analysis - Polysynaptic communication capacity and functional specialization |** (a) Left: Brain map of mean standardized polysynaptic path length from each node to the rest of the network, with red denoting a greater integration of the node within the network and yellow denoting a lower integration. Right: Significant anticorrelations ($p_{\text{spin}} < .05$ in grey; Bonferroni corrected, $\boldsymbol{\alpha = .05}$ in green) between node-wise mean standardized path length and Neurosynth functional activation maps associated to higher-order cognitive functions are replicated in polysynaptic communication pathways. (b) Same as (a) but for polysynaptic communication pathways standardized using a geometry-preserving null model.

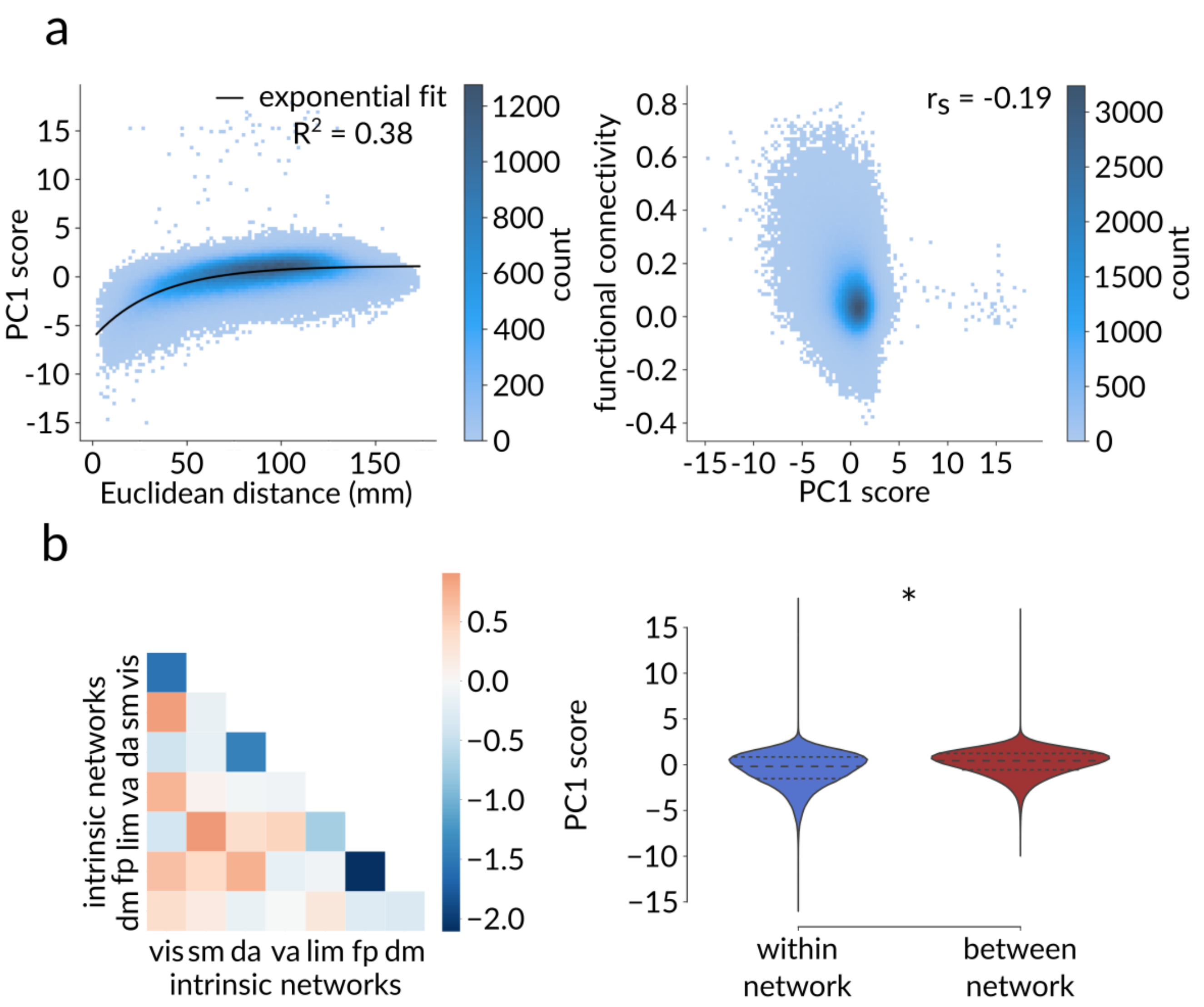

Figure S9. **Sensitivity analysis - Polysynaptic communication pathways from multiple communication models** | The PC1 aggregate communication score recapitulates the results obtained using standardized shortest path length even if we circumscribe the analyses to PC1 scores of node pairs separated by more than one synapse. (a) Left: Growth of the PC1 score as a function of Euclidean distance. The black line corresponds to the fitted exponential $\boldsymbol{y = -7.49e^{-0.03x} + 1.12}$. Right: Negative Spearman correlation between functional connectivity and PC1 score ($r_s = -.19, p \approx 0$). (b) Left: Heatmap of the mean PC1 score across node pairs belonging to the same intrinsic network (diagonal) and to different intrinsic networks (off-diagonal). Right: Significantly lower within-network than between-network PC1 score ($p_{\text{spin}} < .001$).

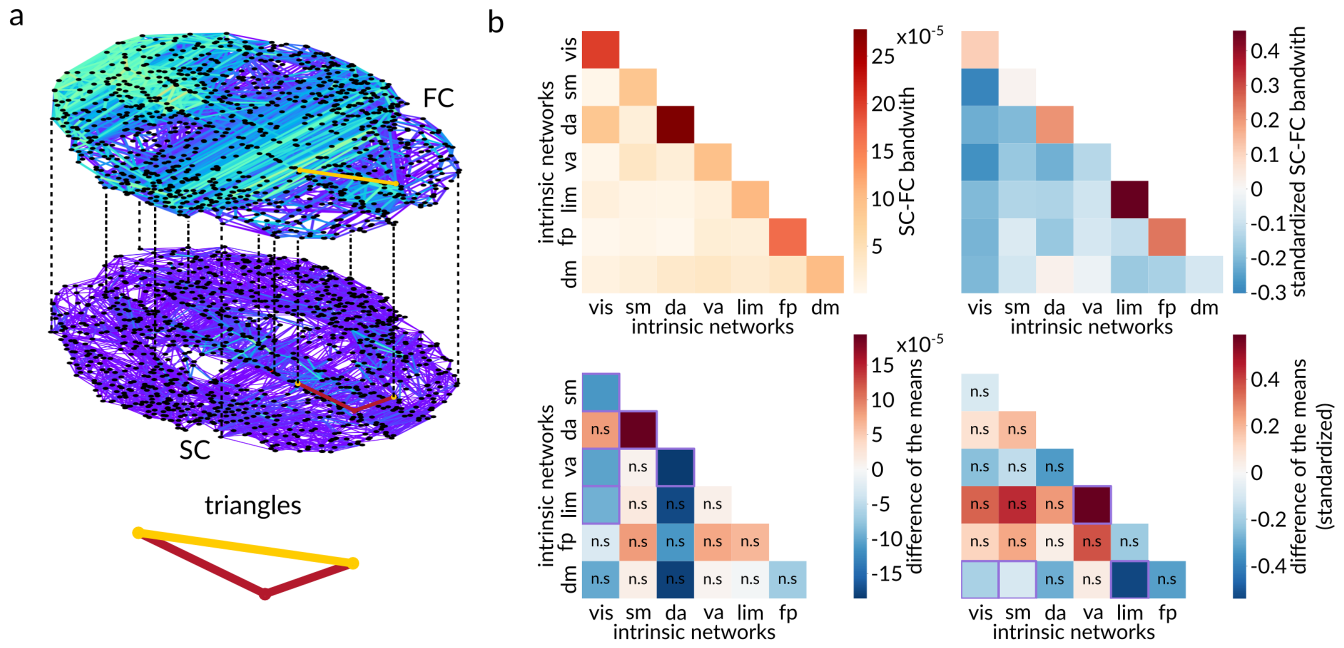

Figure S10. **Sensitivity analysis - higher-order connectivity** | (a) 2-layer multiplex of cortical connectivity. Edges are showed in red in the lower structural connectivity (SC) layer, whereas they appear in yellow in the upper functional connectivity (FC) layer. Dashed lines represent pseudo-edges connecting the two layers to close structural pathways by an FC edge, forming SC-FC polygons. (b) Top: Heatmap of the mean empirical (left) and standardized (right) SC-FC triangles bandwidth across node pairs of the FC layer belonging to the same intrinsic network (diagonal) and to different intrinsic networks (off-diagonal). Bottom: Heatmap of the pairwise differences of the means among Yeo intrinsic networks, calculated as the mean value of the network on the y-axis minus the mean value of the network on the x-axis, with the mean value corresponding to the mean empirical (left) and standardized (right) SC-FC triangles bandwidth across node pairs of the FC layer belonging to the same network (diagonal elements of the top heatmap). A purple square indicates significant difference of the means based on network label permutation using spatial autocorrelation-preserving null models (Bonferroni corrected, $\alpha = .05$), whereas "n.s." denotes not significant differences. The dorsal attention network displays a consistently higher internal SC-FC triangles bandwidth compared to other networks, whereas the somatomotor network exhibits a consistently lower SC-FC triangles bandwidth. These results are not maintained when standardizing SC-FC bandwidth, with the limbic network now showing the highest triangles bandwidth, and the ventral attention network showing the lowest.
